# Intrapericardial Giant Lipoma Displacing the Heart

**DOI:** 10.5402/2011/243637

**Published:** 2011-05-29

**Authors:** C. M. Steger

**Affiliations:** Department of Pathology, Innsbruck Medical University, Müllerstraße 44, 6020 Innsbruck, Austria

## Abstract

Despite their benign character, intrapericardial lipomas can cause life-threatening complications by rapid growth. This paper presents a case of an intrapericardial lipoma in an almost asymptomatic 41-year-old female patient only suffering from mild dyspnoea on exertion. The tumour was found incidentally by chest X-ray. Echocardiographic examination and a CT scan of the thorax revealed a 16 × 14 × 12 cm lipomatous tumour mass highly suspective of a lipoma. Histological examination of excised tumour specimens confirmed the diagnosis of a lipoma. The patient is currently asymptomatic and has not presented with evidence of recurrence at the 6-month followup.

## 1. Introduction

Cardiac tumors are rare, and lipomas are the least commonly encountered, constituting only 14% of all benign heart tumors [1] and 10% of all neoplasms of the heart. Cardiac lipomas are 50 times less common than myxomas and usually present in combination with lipomas of other organs. These tumours are often of asymptomatic nature and usually detected incidentally, mostly during autopsies. In symptomatic patients, the diagnosis can easily be made by echocardiography, computed tomography, or magnetic resonance imaging. Although lipoma is a benign tumour entity, this benign pathology can lead to compression of cardiac chambers and cause life-threatening complications in an asymptomatic patient when the tumor increases in size.

Etiology of lipomas remains uncertain; an association with gene rearrangements of chromosome 12 has been established in cases of solitary lipomas with an abnormality in the HMGA2-LPP fusion gene [2].

The majority of lipomas occur in the upper half of the body, particularly the trunk and neck, but they can develop in any other side including the hand and heart. There are about 120 lipomas for every liposarcoma. These tumors are typically found in adult patients in the fifth or sixth decade of life but can affect patients of all ages and both sexes [1, 3, 4]. Most reported cases of cardiac lipomas are described as single lesions; however, multiple lipomas have been reported in patients with congenital heart defects, tuberous sclerosis, and rarely in an otherwise normal heart [4–6]. Although they usually do not cause symptoms, intracavitary lesions can manifest with dyspnoea secondary to blood flow obstruction. In addition, involvement of the cardiac conduction system may result in arrhythmias [1, 4, 6]. 

Grossly, lipomas consist of bright yellow fat separated by fine fibrous trabeculae. Microscopically, they are composed of mature adipose tissue with no cellular atypia. Areas of fat necrosis, infarct, and calcification may be present. Morphologic variations of lipomas include fibrolipoma, myxolipoma, chondroid lipoma, myolipoma, spindle cell lipoma, pleomorphic lipoma, and angiolipoma. 

## 2. Case Report

**Figure 1:**
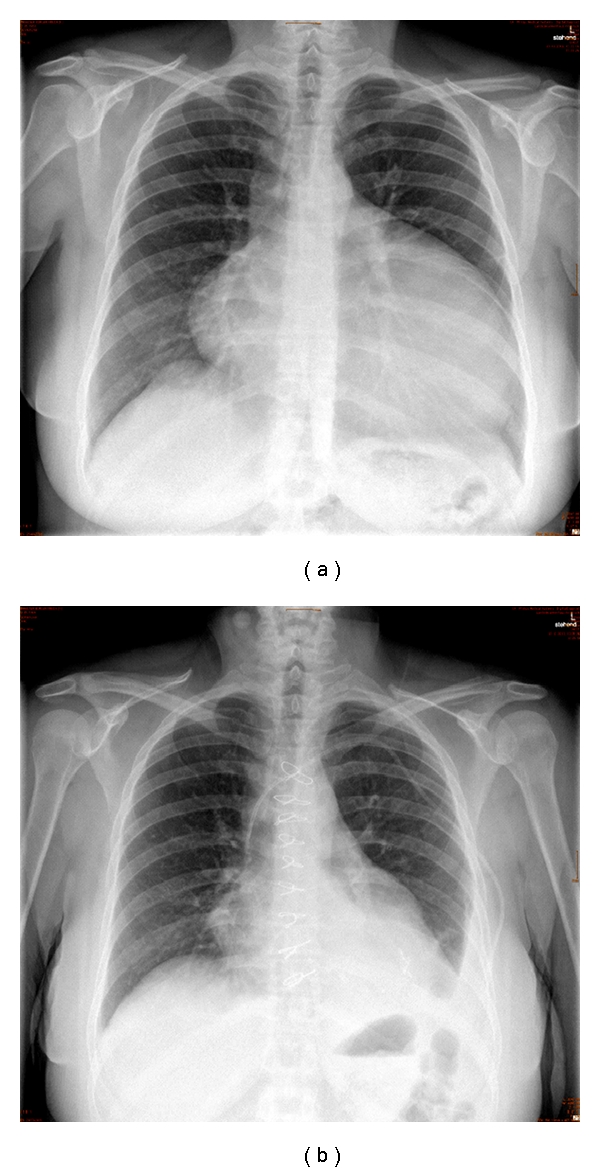
(a) Preoperative chest X-ray with enlargement of the cardiac silhouette. (b) Postoperative chest X-ray after removing the tumour mass.

An almost asymptomatic 41-year-old woman suffering from mild dyspnoea on exertion consulted her physician for the yearly checkup. The physical examination was completely unremarkable, all laboratory testings were within normal limits, and the ECG showed sinus rhythm; only the chest X-ray revealed an enlargement of the cardiac silhouette (Figure 1).

**Figure 2:**
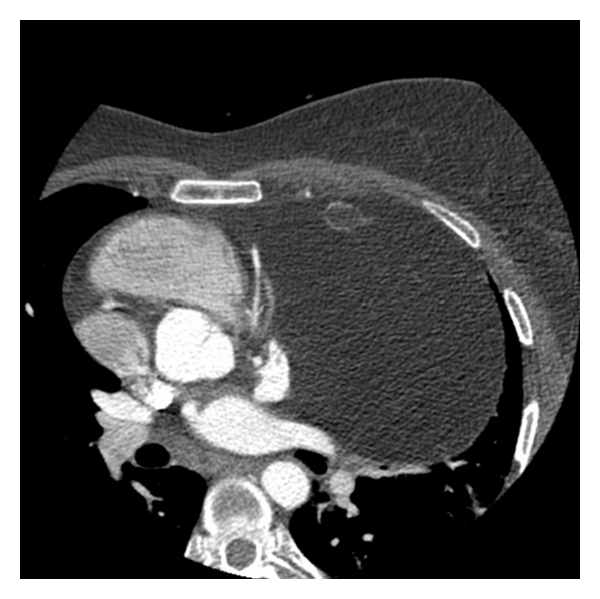
CT of the thorax with the left-sided lipomatous mass displacing the heart to the right hemithorax.

Following echocardiographic examination and contrast-enhanced CT scan of the thorax revealed a lipomatous mass, measuring 16 × 14 × 12 cm, located on the left side of the heart with blood supply from the left coronary artery, highly suspective of a lipoma (Figure 2). Within this mass, a dense calcification (3.5 × 1.5 cm in diameter) in the cranioventral part of the tumour was seen. This large mass caused a marked shift in the midline structures, displacing the heart to the right hemithorax. 

In October 2010, the patient underwent open heart surgery via median sternotomy without cardiopulmonary support. The pericardium was opened on the left side and revealed the left atrium and ventricle pushed to the right side by a lipomatous tumor. The lipoma had a stalk measuring about 5 cm in diameter connected to the diaphragmatic surface of the left ventricle, but it did not adhere to the pericardium. The tumour mass was completely removed, hemostasis was achieved, and the chest was closed after placing one chest tube in the pericardium. The postoperative course was uneventful and the patient was discharged from hospital on the 6th postoperative day. The patient is currently asymptomatic and has not presented with evidence of recurrence at the 6-month followup. 

**Figure 3:**
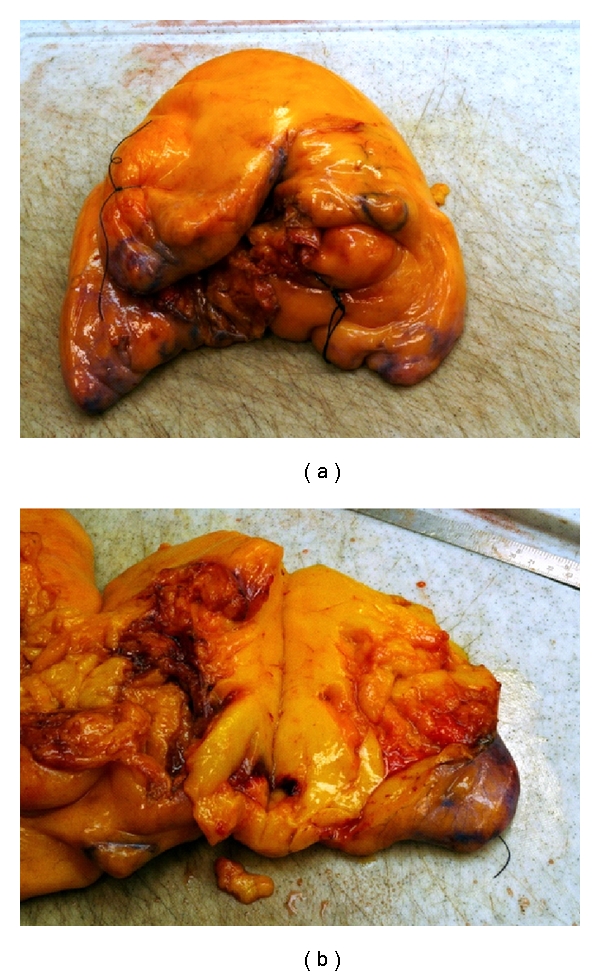
The yellowish lipoma mass with the lobulated surface and fibrous capsule.

**Figure 4:**
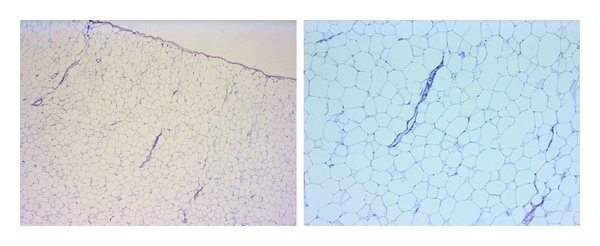
Histological examination of the lipoma revealed mature adipocytes accompanied by some small vessels and limited by a collagenous capsule. (H&E staining; left—original magnification ×4, right—original magnification ×10).

Gross examination revealed a homogenous yellowish vascularized tumour mass with a smooth lobulated surface limited by a fibrous capsule (Figure 3). Histologically, the excised specimen showed a fatty structure of mature benign fat with some areas of increased vascularity (Figure 4). 

## 3. Discussion

Since the first successful surgical removal of an intrapericardial lipoma by Maurer [7] in 1952, further sporadic cases have been reported in the world literature. Although lipomas are benign and slow growing, surgical removal of all cardiac lipomas is necessary to prevent symptoms that could lead to tumour compression of the heart. 

Patients suffering from cardiac lipomas may remain asymptomatic for a long time. Occasionally, large subpericardial lipomas may cause anginal pain by compressing the coronary arteries, dyspnoea, embolism, and atrial and ventricular arrhythmias. Other symptoms that have been described are palpitations, ECG changes, heart valve dysfunction, flow obstruction in the inferior or superior caval vein, and cardiac failure as well as phrenic nerve lesions.

Most of cardiac lipomas are subendocardial or epicardial, and only 25% are found in the myocardium. They usually originate from epicardial fat tissue and grow into the pericardial sac. The most common intracardiac localisation is in the right atrium [8–10] with a wide peduncle originating either from the septal wall or atrial roof. They have also been described in the pericardium [11, 12], in the left atrium [10, 13, 14], on the tricuspid valve [15, 16], on the mitral valve [17], in the right [10] and left ventricles [18], in the ventricular septum [19], and even in both ventricular cavities [20].

The initial investigation in a patient with suspected intrapericardial lipoma should be echocardiography which will usually define the extent and position of the mass. Computed tomography and MRI are the investigations of choice in the demonstration of lipomatous tumours, and both enable differentiation of lipoma from liposarcoma. Additionally, coronary arteriography may be helpful by defining the coronary anatomy and delineating a possible arterial supply originating from the left or right coronary, giving important information to the operating surgeon. Not only radiologically but also histologically, lipomas must be differentiated from liposarcomas, especially from well-differentiated types, which have a predilection for local recurrence and can metastasize. Histologically, cardiac lipomas are largely composed of mature adipocytes, which are usually limited by a collagenous capsule. Well-differentiated liposarcomas usually contain a predominance of mature fat cells with relatively few, widely scattered lipoblasts. A misdiagnosis of lipoma can result from inadequate sampling of the tissue. 

In the case of lipomas, complete surgical excision with the capsule is advocated to prevent local recurrence, whether the lipoma is subcutaneous or intracardiac in origin. For liposarcomas, radiation therapy may be a valuable adjunct to surgery, especially in those of the myxoid variant. The use of chemotherapy in liposarcomas remains experimental.

Cardiac lipomas can be excised with low morbidity and excellent long-term results [21, 22]. Intraoperatively, it is important to excise all of the tumour with the pedicle to prevent recurrence of the tumour, but the rate of lipoma recurrence after total and subtotal resection is very low [10–12, 23]. 

## 4. Conclusion

In conclusion, the majority of intrapericardial lipomas are mostly asymptomatic due to their slow growth. But surgical resection is necessary to prevent tumour compression syndromes of the heart and finally to exclude a malignant process by histological examination of excised specimens.
